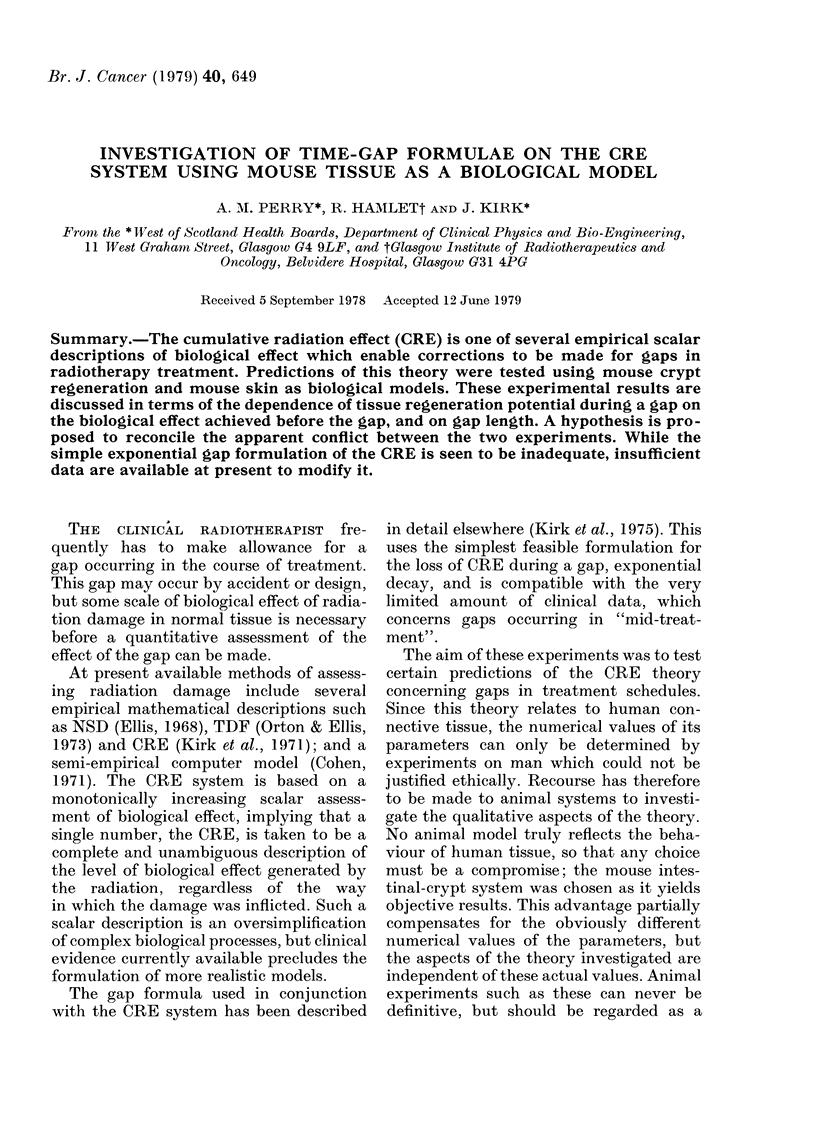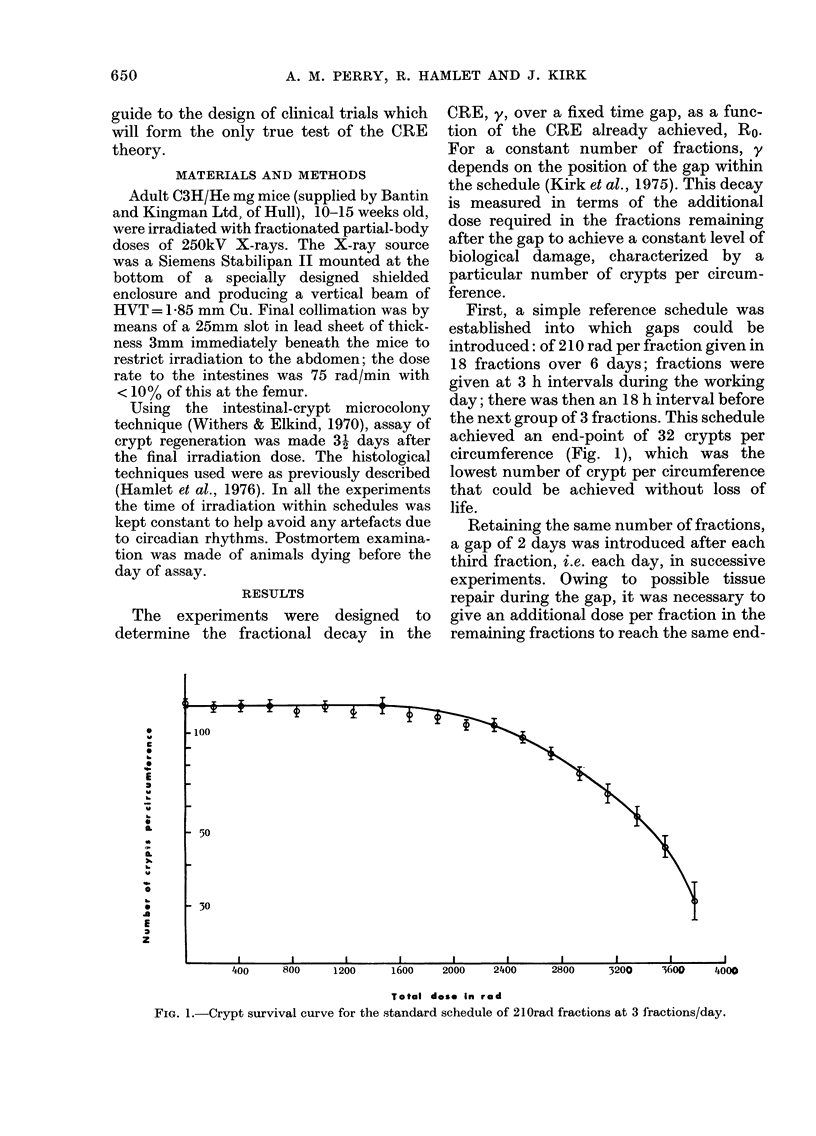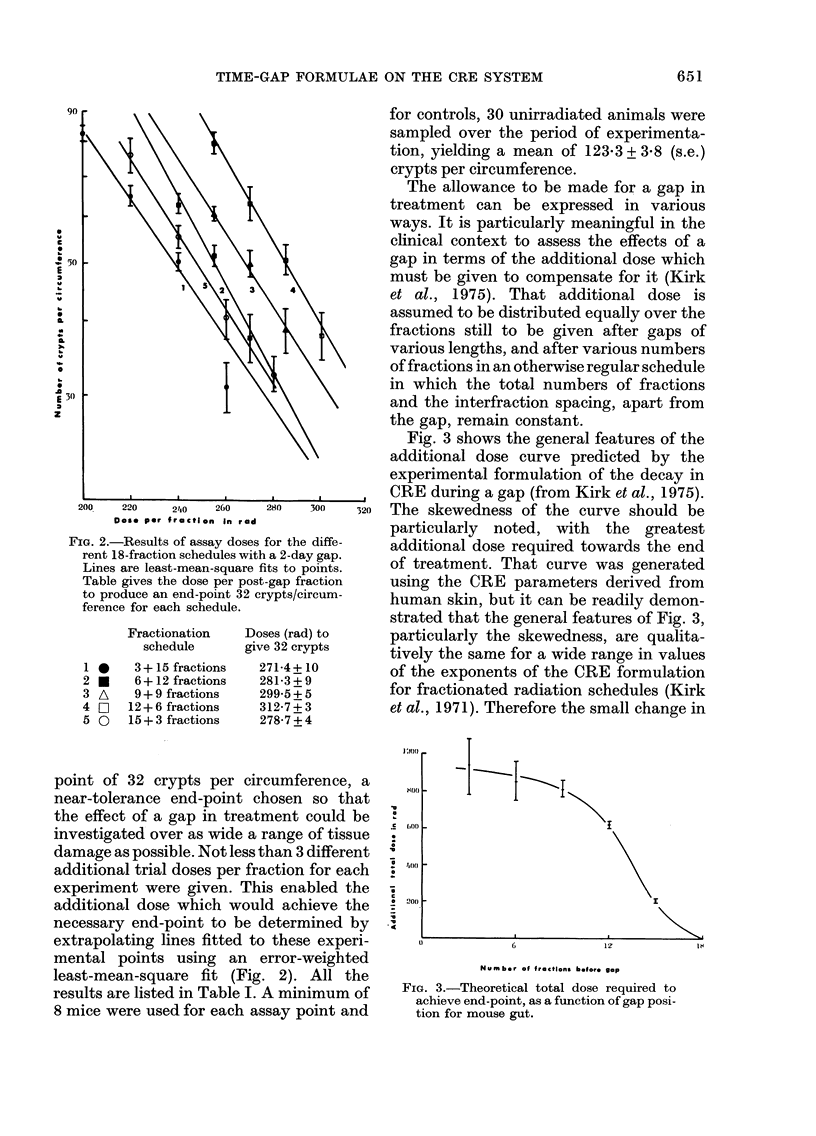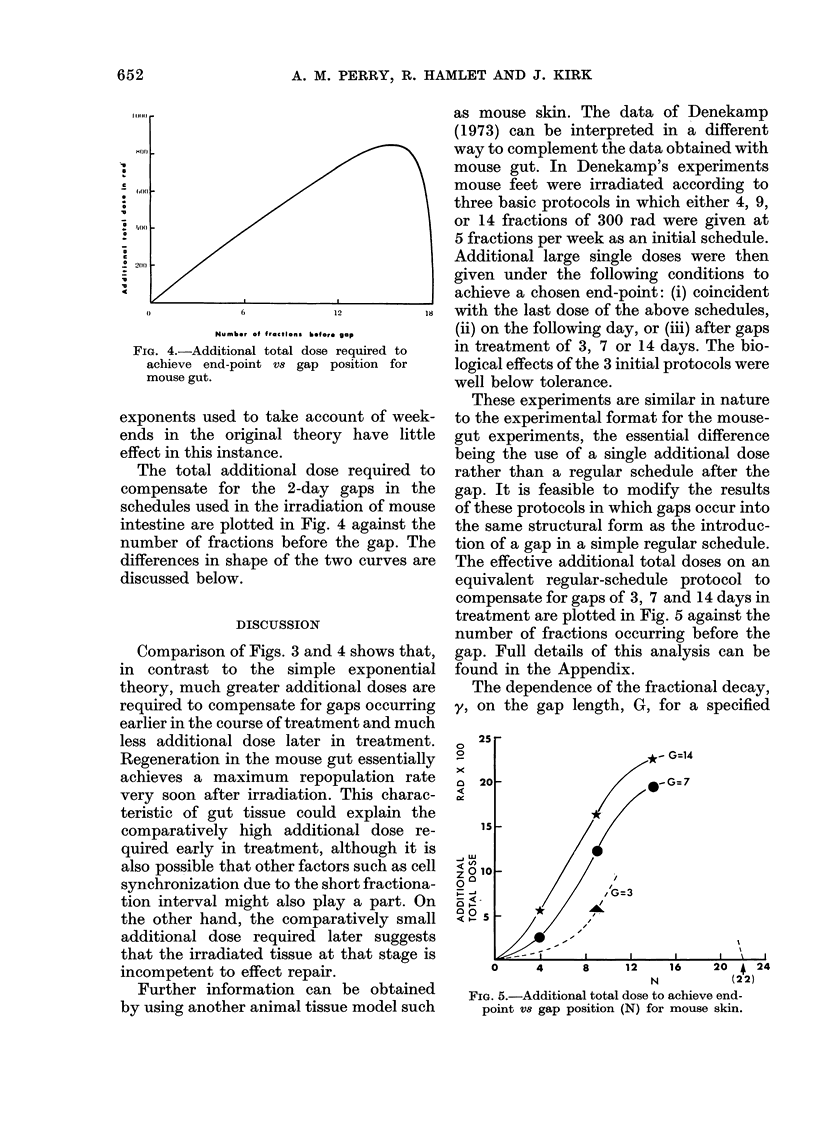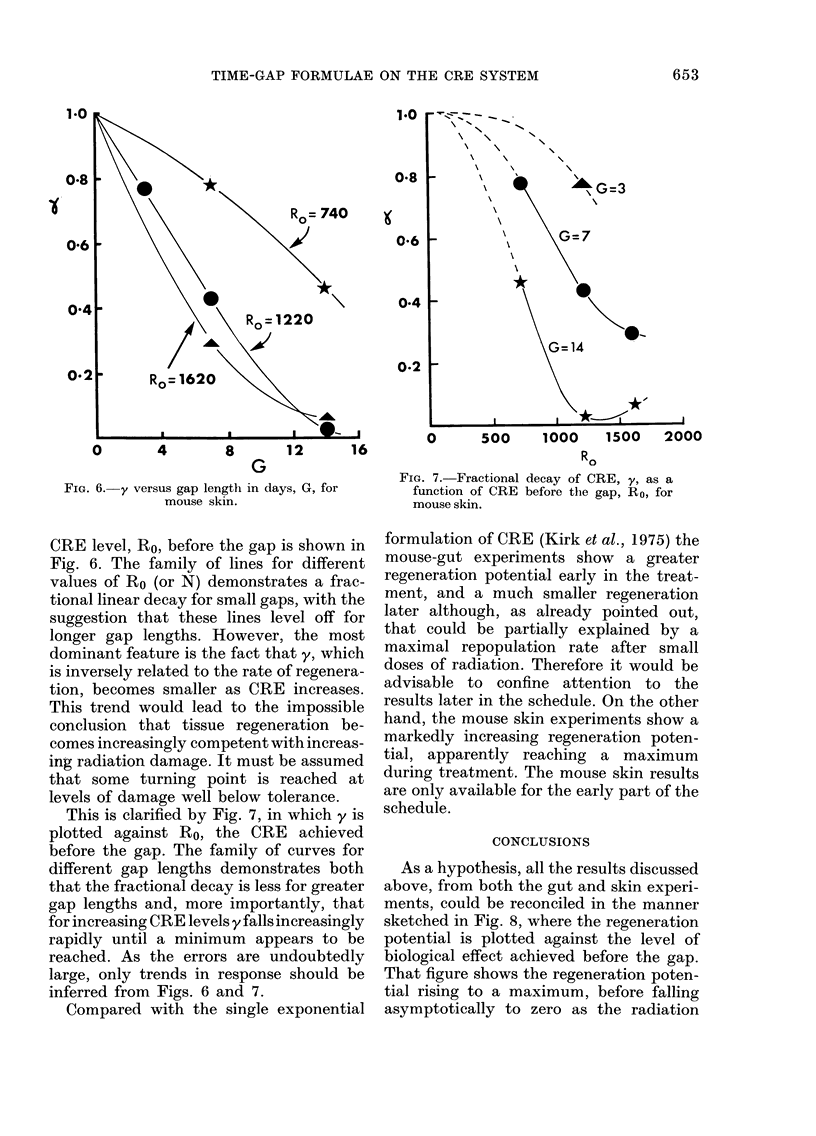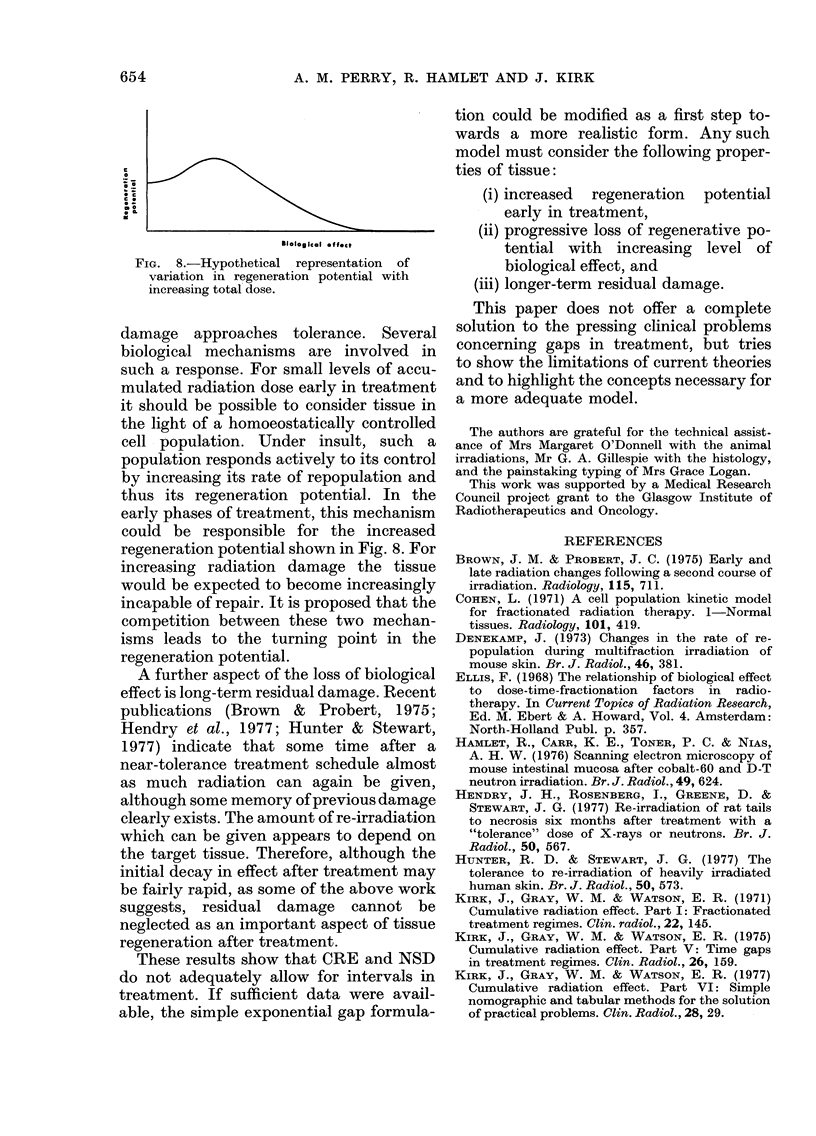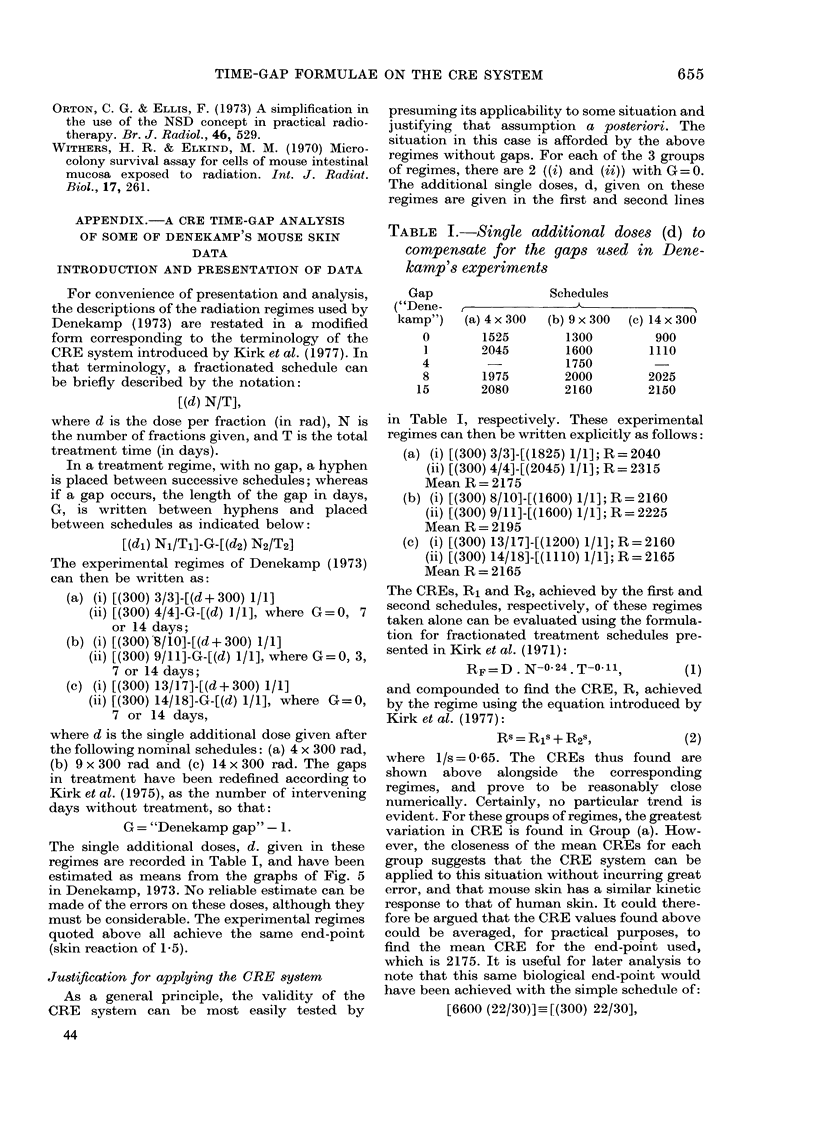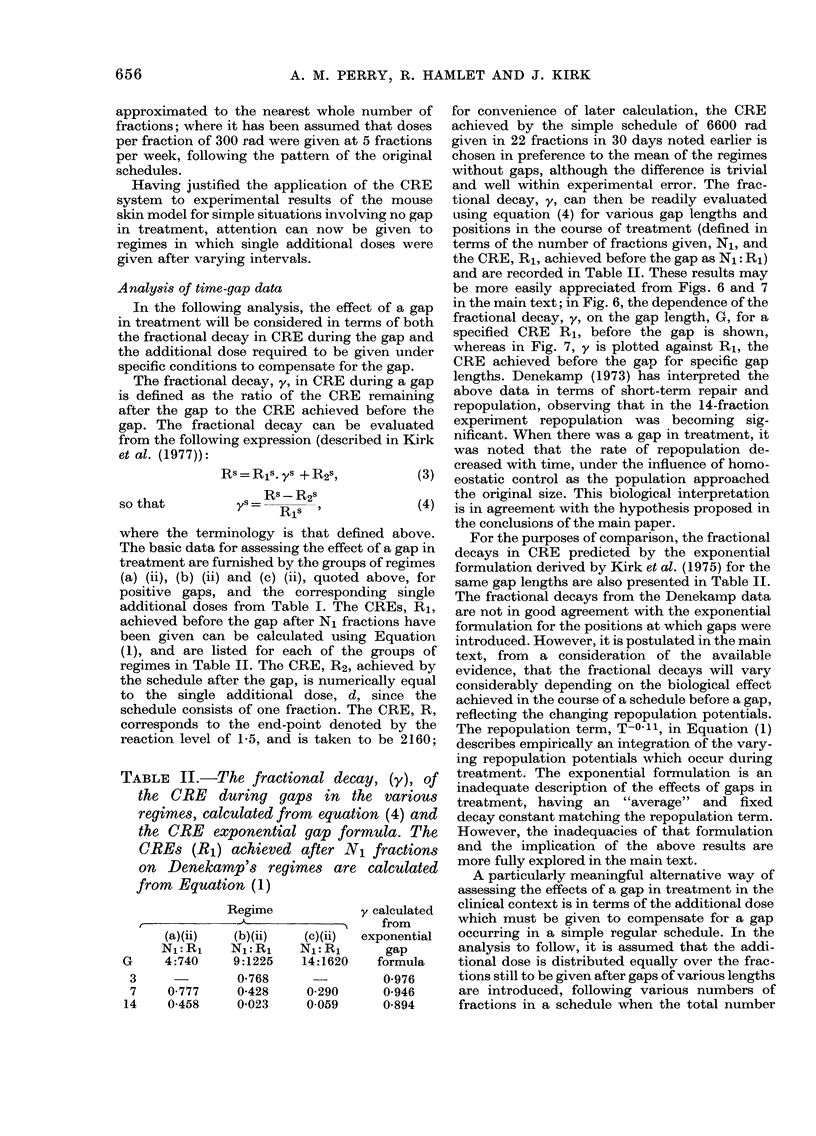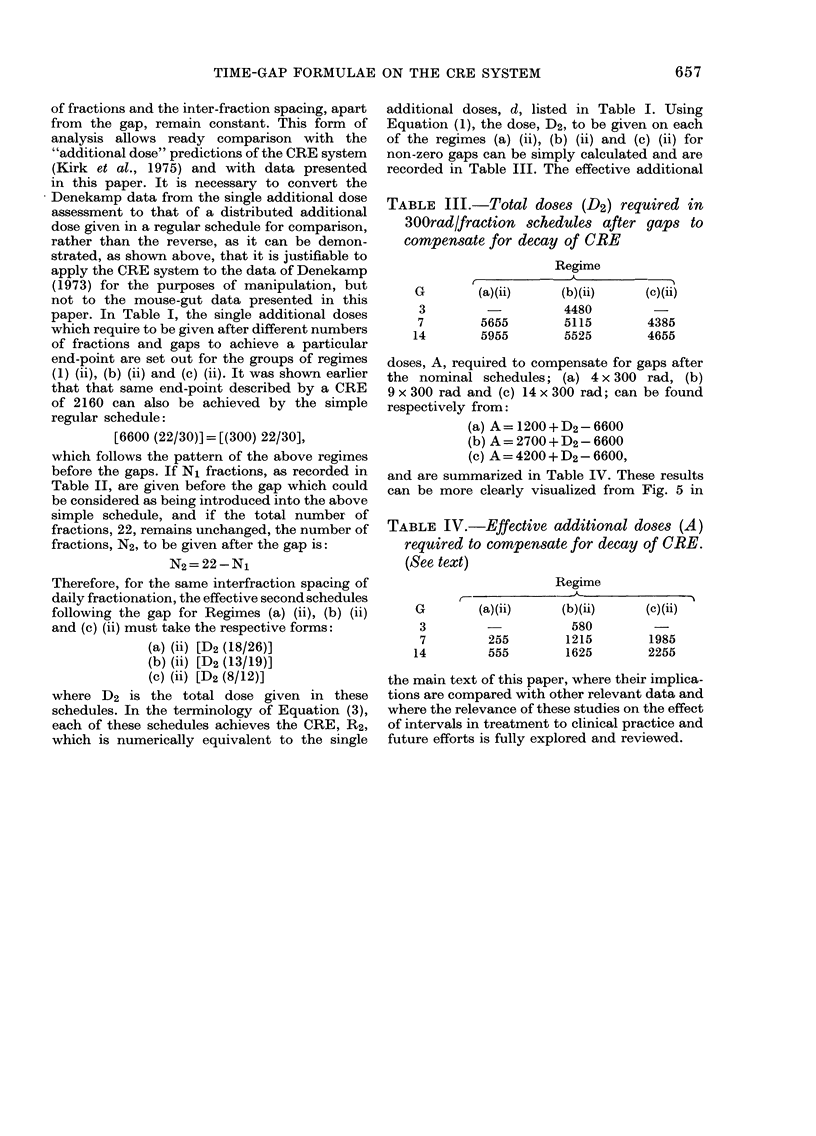# Investigation of time-gap formulae on the CRE system using mouse tissue as a biological model.

**DOI:** 10.1038/bjc.1979.231

**Published:** 1979-10

**Authors:** A. M. Perry, R. Hamlet, J. Kirk

## Abstract

The cumulative radiation effect (CRE) is one of several empirical scalar descriptions of biological effect which enable corrections to be made for gaps in radiotherapy treatment. Predictions of this theory were tested using mouse crypt regeneration and mouse skin as biological models. These experimental results are discussed in terms of the dependence of tissue regeneration potential during a gap on the biological effect achieved before the gap, and on gap length. A hypothesis is proposed to reconcile the apparent conflict between the two experiments. While the simple exponential gap formulation of the CRE is seen to be inadequate, insufficient data are available at present to modify it.


*Br. J. Cancer* (1979) **40**, 649

### INVESTIGATION OF TIME-GAP FORMULAE ON THE CRE SYSTEM USING MOUSE TISSUE AS A BIOLOGICAL MODEL

A. M. PERRY*, R. HAMLET† AND J. KIRK*

*From the *West of Scotland Health Boards, Department of Clinical Physics and Bio-Engineering, 11 West Graham Street, Glasgow G4 9LF, and †Glasgow Institute of Radiotherapeutics and Oncology, Belvidere Hospital, Glasgow G31 4PG*

Received 5 September 1978 Accepted 12 June 1979

**Summary.—The cumulative radiation effect (CRE) is one of several empirical scalar descriptions of biological effect which enable corrections to be made for gaps in radiotherapy treatment. Predictions of this theory were tested using mouse crypt regeneration and mouse skin as biological models. These experimental results are discussed in terms of the dependence of tissue regeneration potential during a gap on the biological effect achieved before the gap, and on gap length. A hypothesis is proposed to reconcile the apparent conflict between the two experiments. While the simple exponential gap formulation of the CRE is seen to be inadequate, insufficient data are available at present to modify it.**

THE CLINICAL RADIOTHERAPIST frequently has to make allowance for a gap occurring in the course of treatment. This gap may occur by accident or design, but some scale of biological effect of radiation damage in normal tissue is necessary before a quantitative assessment of the effect of the gap can be made.

At present available methods of assessing radiation damage include several empirical mathematical descriptions such as NSD (Ellis, 1968), TDF (Orton & Ellis, 1973) and CRE (Kirk *et al.*, 1971); and a semi-empirical computer model (Cohen, 1971). The CRE system is based on a monotonically increasing scalar assessment of biological effect, implying that a single number, the CRE, is taken to be a complete and unambiguous description of the level of biological effect generated by the radiation, regardless of the way in which the damage was inflicted. Such a scalar description is an oversimplification of complex biological processes, but clinical evidence currently available precludes the formulation of more realistic models.

The gap formula used in conjunction with the CRE system has been described in detail elsewhere (Kirk *et al.*, 1975). This uses the simplest feasible formulation for the loss of CRE during a gap, exponential decay, and is compatible with the very limited amount of clinical data, which concerns gaps occurring in "mid-treatment".

The aim of these experiments was to test certain predictions of the CRE theory concerning gaps in treatment schedules. Since this theory relates to human connective tissue, the numerical values of its parameters can only be determined by experiments on man which could not be justified ethically. Recourse has therefore to be made to animal systems to investigate the qualitative aspects of the theory. No animal model truly reflects the behaviour of human tissue, so that any choice must be a compromise; the mouse intestinal-crypt system was chosen as it yields objective results. This advantage partially compensates for the obviously different numerical values of the parameters, but the aspects of the theory investigated are independent of these actual values. Animal experiments such as these can never be definitive, but should be regarded as a guide to the design of clinical trials which will form the only true test of the CRE theory.

MATERIALS AND METHODS

Adult C3H/He mg mice (supplied by Bantin and Kingman Ltd, of Hull), 10–15 weeks old, were irradiated with fractionated partial-body doses of 250kV X-rays. The X-ray source was a Siemens Stabilipan II mounted at the bottom of a specially designed shielded enclosure and producing a vertical beam of HVT = 1·85 mm Cu. Final collimation was by means of a 25mm slot in lead sheet of thickness 3mm immediately beneath the mice to restrict irradiation to the abdomen; the dose rate to the intestines was 75 rad/min with < 10% of this at the femur.

Using the intestinal-crypt microcolony technique (Withers & Elkind, 1970), assay of crypt regeneration was made 3½ days after the final irradiation dose. The histological techniques used were as previously described (Hamlet *et al.*, 1976). In all the experiments the time of irradiation within schedules was kept constant to help avoid any artefacts due to circadian rhythms. Postmortem examination was made of animals dying before the day of assay.

RESULTS

The experiments were designed to determine the fractional decay in the CRE, $\gamma$, over a fixed time gap, as a function of the CRE already achieved, $R_0$. For a constant number of fractions, $\gamma$ depends on the position of the gap within the schedule (Kirk *et al.*, 1975). This decay is measured in terms of the additional dose required in the fractions remaining after the gap to achieve a constant level of biological damage, characterized by a particular number of crypts per circumference.

First, a simple reference schedule was established into which gaps could be introduced: of 210 rad per fraction given in 18 fractions over 6 days; fractions were given at 3 h intervals during the working day; there was then an 18 h interval before the next group of 3 fractions. This schedule achieved an end-point of 32 crypts per circumference (Fig. 1), which was the lowest number of crypt per circumference that could be achieved without loss of life.

Retaining the same number of fractions, a gap of 2 days was introduced after each third fraction, *i.e.* each day, in successive experiments. Owing to possible tissue repair during the gap, it was necessary to give an additional dose per fraction in the remaining fractions to reach the same end-

FIG. 1.—Crypt survival curve for the standard schedule of 210rad fractions at 3 fractions/day.

FIG. 2.—Results of assay doses for the different 18-fraction schedules with a 2-day gap. Lines are least-mean-square fits to points. Table gives the dose per post-gap fraction to produce an end-point 32 crypts/circumference for each schedule.

| | Fractionation schedule | Doses (rad) to give 32 crypts |
|---|---|---|
| 1 ● | 3+15 fractions | 271·4±10 |
| 2 ■ | 6+12 fractions | 281·3±9 |
| 3 △ | 9+9 fractions | 299·5±5 |
| 4 □ | 12+6 fractions | 312·7±3 |
| 5 ○ | 15+3 fractions | 278·7±4 |

point of 32 crypts per circumference, a near-tolerance end-point chosen so that the effect of a gap in treatment could be investigated over as wide a range of tissue damage as possible. Not less than 3 different additional trial doses per fraction for each experiment were given. This enabled the additional dose which would achieve the necessary end-point to be determined by extrapolating lines fitted to these experimental points using an error-weighted least-mean-square fit (Fig. 2). All the results are listed in Table I. A minimum of 8 mice were used for each assay point and for controls, 30 unirradiated animals were sampled over the period of experimentation, yielding a mean of 123·3 ± 3·8 (s.e.) crypts per circumference.

The allowance to be made for a gap in treatment can be expressed in various ways. It is particularly meaningful in the clinical context to assess the effects of a gap in terms of the additional dose which must be given to compensate for it (Kirk *et al.*, 1975). That additional dose is assumed to be distributed equally over the fractions still to be given after gaps of various lengths, and after various numbers of fractions in an otherwise regular schedule in which the total numbers of fractions and the interfraction spacing, apart from the gap, remain constant.

Fig. 3 shows the general features of the additional dose curve predicted by the experimental formulation of the decay in CRE during a gap (from Kirk *et al.*, 1975). The skewedness of the curve should be particularly noted, with the greatest additional dose required towards the end of treatment. That curve was generated using the CRE parameters derived from human skin, but it can be readily demonstrated that the general features of Fig. 3, particularly the skewedness, are qualitatively the same for a wide range in values of the exponents of the CRE formulation for fractionated radiation schedules (Kirk *et al.*, 1971). Therefore the small change in

FIG. 3.—Theoretical total dose required to achieve end-point, as a function of gap position for mouse gut.

FIG. 4.—Additional total dose required to achieve end-point *vs* gap position for mouse gut.

exponents used to take account of weekends in the original theory have little effect in this instance.

The total additional dose required to compensate for the 2-day gaps in the schedules used in the irradiation of mouse intestine are plotted in Fig. 4 against the number of fractions before the gap. The differences in shape of the two curves are discussed below.

### DISCUSSION

Comparison of Figs. 3 and 4 shows that, in contrast to the simple exponential theory, much greater additional doses are required to compensate for gaps occurring earlier in the course of treatment and much less additional dose later in treatment. Regeneration in the mouse gut essentially achieves a maximum repopulation rate very soon after irradiation. This characteristic of gut tissue could explain the comparatively high additional dose required early in treatment, although it is also possible that other factors such as cell synchronization due to the short fractionation interval might also play a part. On the other hand, the comparatively small additional dose required later suggests that the irradiated tissue at that stage is incompetent to effect repair.

Further information can be obtained by using another animal tissue model such as mouse skin. The data of Denekamp (1973) can be interpreted in a different way to complement the data obtained with mouse gut. In Denekamp's experiments mouse feet were irradiated according to three basic protocols in which either 4, 9, or 14 fractions of 300 rad were given at 5 fractions per week as an initial schedule. Additional large single doses were then given under the following conditions to achieve a chosen end-point: (i) coincident with the last dose of the above schedules, (ii) on the following day, or (iii) after gaps in treatment of 3, 7 or 14 days. The biological effects of the 3 initial protocols were well below tolerance.

These experiments are similar in nature to the experimental format for the mouse-gut experiments, the essential difference being the use of a single additional dose rather than a regular schedule after the gap. It is feasible to modify the results of these protocols in which gaps occur into the same structural form as the introduction of a gap in a simple regular schedule. The effective additional total doses on an equivalent regular-schedule protocol to compensate for gaps of 3, 7 and 14 days in treatment are plotted in Fig. 5 against the number of fractions occurring before the gap. Full details of this analysis can be found in the Appendix.

The dependence of the fractional decay, $\gamma$, on the gap length, G, for a specified

FIG. 5.—Additional total dose to achieve end-point *vs* gap position (N) for mouse skin.

FIG. 6.—$\gamma$ versus gap length in days, G, for mouse skin.

FIG. 7.—Fractional decay of CRE, $\gamma$, as a function of CRE before the gap, $R_0$, for mouse skin.

CRE level, $R_0$, before the gap is shown in Fig. 6. The family of lines for different values of $R_0$ (or N) demonstrates a fractional linear decay for small gaps, with the suggestion that these lines level off for longer gap lengths. However, the most dominant feature is the fact that $\gamma$, which is inversely related to the rate of regeneration, becomes smaller as CRE increases. This trend would lead to the impossible conclusion that tissue regeneration becomes increasingly competent with increasing radiation damage. It must be assumed that some turning point is reached at levels of damage well below tolerance.

This is clarified by Fig. 7, in which $\gamma$ is plotted against $R_0$, the CRE achieved before the gap. The family of curves for different gap lengths demonstrates both that the fractional decay is less for greater gap lengths and, more importantly, that for increasing CRE levels $\gamma$ falls increasingly rapidly until a minimum appears to be reached. As the errors are undoubtedly large, only trends in response should be inferred from Figs. 6 and 7.

Compared with the single exponential formulation of CRE (Kirk *et al.*, 1975) the mouse-gut experiments show a greater regeneration potential early in the treatment, and a much smaller regeneration later although, as already pointed out, that could be partially explained by a maximal repopulation rate after small doses of radiation. Therefore it would be advisable to confine attention to the results later in the schedule. On the other hand, the mouse skin experiments show a markedly increasing regeneration potential, apparently reaching a maximum during treatment. The mouse skin results are only available for the early part of the schedule.

### CONCLUSIONS

As a hypothesis, all the results discussed above, from both the gut and skin experiments, could be reconciled in the manner sketched in Fig. 8, where the regeneration potential is plotted against the level of biological effect achieved before the gap. That figure shows the regeneration potential rising to a maximum, before falling asymptotically to zero as the radiation

FIG. 8.—Hypothetical representation of variation in regeneration potential with increasing total dose.

damage approaches tolerance. Several biological mechanisms are involved in such a response. For small levels of accumulated radiation dose early in treatment it should be possible to consider tissue in the light of a homoeostatically controlled cell population. Under insult, such a population responds actively to its control by increasing its rate of repopulation and thus its regeneration potential. In the early phases of treatment, this mechanism could be responsible for the increased regeneration potential shown in Fig. 8. For increasing radiation damage the tissue would be expected to become increasingly incapable of repair. It is proposed that the competition between these two mechanisms leads to the turning point in the regeneration potential.

A further aspect of the loss of biological effect is long-term residual damage. Recent publications (Brown & Probert, 1975; Hendry *et al.*, 1977; Hunter & Stewart, 1977) indicate that some time after a near-tolerance treatment schedule almost as much radiation can again be given, although some memory of previous damage clearly exists. The amount of re-irradiation which can be given appears to depend on the target tissue. Therefore, although the initial decay in effect after treatment may be fairly rapid, as some of the above work suggests, residual damage cannot be neglected as an important aspect of tissue regeneration after treatment.

These results show that CRE and NSD do not adequately allow for intervals in treatment. If sufficient data were available, the simple exponential gap formulation could be modified as a first step towards a more realistic form. Any such model must consider the following properties of tissue:

(i) increased regeneration potential early in treatment,
(ii) progressive loss of regenerative potential with increasing level of biological effect, and
(iii) longer-term residual damage.

This paper does not offer a complete solution to the pressing clinical problems concerning gaps in treatment, but tries to show the limitations of current theories and to highlight the concepts necessary for a more adequate model.

The authors are grateful for the technical assistance of Mrs Margaret O'Donnell with the animal irradiations, Mr G. A. Gillespie with the histology, and the painstaking typing of Mrs Grace Logan.

This work was supported by a Medical Research Council project grant to the Glasgow Institute of Radiotherapeutics and Oncology.

### REFERENCES

BROWN, J. M. & PROBERT, J. C. (1975) Early and late radiation changes following a second course of irradiation. *Radiology*, **115**, 711.

COHEN, L. (1971) A cell population kinetic model for fractionated radiation therapy. 1—Normal tissues. *Radiology*, **101**, 419.

DENEKAMP, J. (1973) Changes in the rate of repopulation during multifraction irradiation of mouse skin. *Br. J. Radiol.*, **46**, 381.

ELLIS, F. (1968) The relationship of biological effect to dose-time-fractionation factors in radiotherapy. In *Current Topics of Radiation Research*, Ed. M. Ebert & A. Howard, Vol. 4. Amsterdam: North-Holland Publ. p. 357.

HAMLET, R., CARR, K. E., TONER, P. C. & NIAS, A. H. W. (1976) Scanning electron microscopy of mouse intestinal mucosa after cobalt-60 and D-T neutron irradiation. *Br. J. Radiol.*, **49**, 624.

HENDRY, J. H., ROSENBERG, I., GREENE, D. & STEWART, J. G. (1977) Re-irradiation of rat tails to necrosis six months after treatment with a "tolerance" dose of X-rays or neutrons. *Br. J. Radiol.*, **50**, 567.

HUNTER, R. D. & STEWART, J. G. (1977) The tolerance to re-irradiation of heavily irradiated human skin. *Br. J. Radiol.*, **50**, 573.

KIRK, J., GRAY, W. M. & WATSON, E. R. (1971) Cumulative radiation effect. Part I: Fractionated treatment regimes. *Clin. radiol.*, **22**, 145.

KIRK, J., GRAY, W. M. & WATSON, E. R. (1975) Cumulative radiation effect. Part V: Time gaps in treatment regimes. *Clin. Radiol.*, **26**, 159.

KIRK, J., GRAY, W. M. & WATSON, E. R. (1977) Cumulative radiation effect. Part VI: Simple nomographic and tabular methods for the solution of practical problems. *Clin. Radiol.*, **28**, 29.

ORTON, C. G. & ELLIS, F. (1973) A simplification in the use of the NSD concept in practical radiotherapy. *Br. J. Radiol.*, **46**, 529.
WITHERS, H. R. & ELKIND, M. M. (1970) Microcolony survival assay for cells of mouse intestinal mucosa exposed to radiation. *Int. J. Radiat. Biol.*, **17**, 261.

### APPENDIX.—A CRE TIME-GAP ANALYSIS OF SOME OF DENEKAMP'S MOUSE SKIN DATA

### INTRODUCTION AND PRESENTATION OF DATA

For convenience of presentation and analysis, the descriptions of the radiation regimes used by Denekamp (1973) are restated in a modified form corresponding to the terminology of the CRE system introduced by Kirk *et al.* (1977). In that terminology, a fractionated schedule can be briefly described by the notation:

$$[(d)\ N/T],$$

where $d$ is the dose per fraction (in rad), N is the number of fractions given, and T is the total treatment time (in days).

In a treatment regime, with no gap, a hyphen is placed between successive schedules; whereas if a gap occurs, the length of the gap in days, G, is written between hyphens and placed between schedules as indicated below:

$$[(d_1)\ N_1/T_1]\text{-G-}[(d_2)\ N_2/T_2]$$

The experimental regimes of Denekamp (1973) can then be written as:

(a) (i) [(300) 3/3]-[($d$ + 300) 1/1]
(ii) [(300) 4/4]-G-[($d$) 1/1], where G = 0, 7 or 14 days;
(b) (i) [(300) 8/10]-[($d$ + 300) 1/1]
(ii) [(300) 9/11]-G-[($d$) 1/1], where G = 0, 3, 7 or 14 days;
(c) (i) [(300) 13/17]-[($d$ + 300) 1/1]
(ii) [(300) 14/18]-G-[($d$) 1/1], where G = 0, 7 or 14 days, where $d$ is the single additional dose given after the following nominal schedules: (a) 4 × 300 rad, (b) 9 × 300 rad and (c) 14 × 300 rad. The gaps in treatment have been redefined according to Kirk *et al.* (1975), as the number of intervening days without treatment, so that:

$$G = \text{"Denekamp gap"} - 1.$$

The single additional doses, $d$. given in these regimes are recorded in Table I, and have been estimated as means from the graphs of Fig. 5 in Denekamp, 1973. No reliable estimate can be made of the errors on these doses, although they must be considerable. The experimental regimes quoted above all achieve the same end-point (skin reaction of 1·5).

### *Justification for applying the CRE system*

As a general principle, the validity of the CRE system can be most easily tested by presuming its applicability to some situation and justifying that assumption *a posteriori*. The situation in this case is afforded by the above regimes without gaps. For each of the 3 groups of regimes, there are 2 ((*i*) and (*ii*)) with G = 0. The additional single doses, d, given on these regimes are given in the first and second lines in Table I, respectively. These experimental regimes can then be written explicitly as follows:

TABLE I.—*Single additional doses* (d) *to compensate for the gaps used in Denekamp's experiments*

| Gap ("Denekamp") | Schedules (a) 4 × 300 | (b) 9 × 300 | (c) 14 × 300 |
|---|---|---|---|
| 0 | 1525 | 1300 | 900 |
| 1 | 2045 | 1600 | 1110 |
| 4 | — | 1750 | — |
| 8 | 1975 | 2000 | 2025 |
| 15 | 2080 | 2160 | 2150 |

(a) (i) [(300) 3/3]-[(1825) 1/1]; R = 2040
(ii) [(300) 4/4]-[(2045) 1/1]; R = 2315
Mean R = 2175
(b) (i) [(300) 8/10]-[(1600) 1/1]; R = 2160
(ii) [(300) 9/11]-[(1600) 1/1]; R = 2225
Mean R = 2195
(c) (i) [(300) 13/17]-[(1200) 1/1]; R = 2160
(ii) [(300) 14/18]-[(1110) 1/1]; R = 2165
Mean R = 2165

The CREs, $R_1$ and $R_2$, achieved by the first and second schedules, respectively, of these regimes taken alone can be evaluated using the formulation for fractionated treatment schedules presented in Kirk *et al.* (1971):

$$R_F = D\,.\,N^{-0\cdot24}\,.\,T^{-0\cdot11}, \qquad (1)$$

and compounded to find the CRE, R, achieved by the regime using the equation introduced by Kirk *et al.* (1977):

$$R^s = R_1{}^s + R_2{}^s, \qquad (2)$$

where $1/s = 0{\cdot}65$. The CREs thus found are shown above alongside the corresponding regimes, and prove to be reasonably close numerically. Certainly, no particular trend is evident. For these groups of regimes, the greatest variation in CRE is found in Group (a). However, the closeness of the mean CREs for each group suggests that the CRE system can be applied to this situation without incurring great error, and that mouse skin has a similar kinetic response to that of human skin. It could therefore be argued that the CRE values found above could be averaged, for practical purposes, to find the mean CRE for the end-point used, which is 2175. It is useful for later analysis to note that this same biological end-point would have been achieved with the simple schedule of:

$$[6600\ (22/30)] \equiv [(300)\ 22/30],$$

approximated to the nearest whole number of fractions; where it has been assumed that doses per fraction of 300 rad were given at 5 fractions per week, following the pattern of the original schedules.

Having justified the application of the CRE system to experimental results of the mouse skin model for simple situations involving no gap in treatment, attention can now be given to regimes in which single additional doses were given after varying intervals.

*Analysis of time-gap data*

In the following analysis, the effect of a gap in treatment will be considered in terms of both the fractional decay in CRE during the gap and the additional dose required to be given under specific conditions to compensate for the gap.

The fractional decay, $\gamma$, in CRE during a gap is defined as the ratio of the CRE remaining after the gap to the CRE achieved before the gap. The fractional decay can be evaluated from the following expression (described in Kirk *et al.* (1977)):

$$R^s = R_1{}^s . \gamma^s + R_2{}^s, \qquad (3)$$

so that

$$\gamma^s = \frac{R^s - R_2{}^s}{R_1{}^s}, \qquad (4)$$

where the terminology is that defined above. The basic data for assessing the effect of a gap in treatment are furnished by the groups of regimes (a) (ii), (b) (ii) and (c) (ii), quoted above, for positive gaps, and the corresponding single additional doses from Table I. The CREs, $R_1$, achieved before the gap after $N_1$ fractions have been given can be calculated using Equation (1), and are listed for each of the groups of regimes in Table II. The CRE, $R_2$, achieved by the schedule after the gap, is numerically equal to the single additional dose, *d*, since the schedule consists of one fraction. The CRE, R, corresponds to the end-point denoted by the reaction level of 1·5, and is taken to be 2160; for convenience of later calculation, the CRE achieved by the simple schedule of 6600 rad given in 22 fractions in 30 days noted earlier is chosen in preference to the mean of the regimes without gaps, although the difference is trivial and well within experimental error. The fractional decay, $\gamma$, can then be readily evaluated using equation (4) for various gap lengths and positions in the course of treatment (defined in terms of the number of fractions given, $N_1$, and the CRE, $R_1$, achieved before the gap as $N_1 : R_1$) and are recorded in Table II. These results may be more easily appreciated from Figs. 6 and 7 in the main text; in Fig. 6, the dependence of the fractional decay, $\gamma$, on the gap length, G, for a specified CRE $R_1$, before the gap is shown, whereas in Fig. 7, $\gamma$ is plotted against $R_1$, the CRE achieved before the gap for specific gap lengths. Denekamp (1973) has interpreted the above data in terms of short-term repair and repopulation, observing that in the 14-fraction experiment repopulation was becoming significant. When there was a gap in treatment, it was noted that the rate of repopulation decreased with time, under the influence of homoeostatic control as the population approached the original size. This biological interpretation is in agreement with the hypothesis proposed in the conclusions of the main paper.

TABLE II.—*The fractional decay, ($\gamma$), of the CRE during gaps in the various regimes, calculated from equation* (4) *and the CRE exponential gap formula. The CREs ($R_1$) achieved after $N_1$ fractions on Denekamp's regimes are calculated from Equation* (1)

| | Regime | | | $\gamma$ calculated from exponential gap formula |
|---|---|---|---|---|
| G | (a)(ii) $N_1 : R_1$ 4:740 | (b)(ii) $N_1 : R_1$ 9:1225 | (c)(ii) $N_1 : R_1$ 14:1620 | |
| 3 | — | 0·768 | — | 0·976 |
| 7 | 0·777 | 0·428 | 0·290 | 0·946 |
| 14 | 0·458 | 0·023 | 0·059 | 0·894 |

For the purposes of comparison, the fractional decays in CRE predicted by the exponential formulation derived by Kirk *et al.* (1975) for the same gap lengths are also presented in Table II. The fractional decays from the Denekamp data are not in good agreement with the exponential formulation for the positions at which gaps were introduced. However, it is postulated in the main text, from a consideration of the available evidence, that the fractional decays will vary considerably depending on the biological effect achieved in the course of a schedule before a gap, reflecting the changing repopulation potentials. The repopulation term, $T^{-0 \cdot 11}$, in Equation (1) describes empirically an integration of the varying repopulation potentials which occur during treatment. The exponential formulation is an inadequate description of the effects of gaps in treatment, having an "average" and fixed decay constant matching the repopulation term. However, the inadequacies of that formulation and the implication of the above results are more fully explored in the main text.

A particularly meaningful alternative way of assessing the effects of a gap in treatment in the clinical context is in terms of the additional dose which must be given to compensate for a gap occurring in a simple regular schedule. In the analysis to follow, it is assumed that the additional dose is distributed equally over the fractions still to be given after gaps of various lengths are introduced, following various numbers of fractions in a schedule when the total number of fractions and the inter-fraction spacing, apart from the gap, remain constant. This form of analysis allows ready comparison with the "additional dose" predictions of the CRE system (Kirk *et al.*, 1975) and with data presented in this paper. It is necessary to convert the Denekamp data from the single additional dose assessment to that of a distributed additional dose given in a regular schedule for comparison, rather than the reverse, as it can be demonstrated, as shown above, that it is justifiable to apply the CRE system to the data of Denekamp (1973) for the purposes of manipulation, but not to the mouse-gut data presented in this paper. In Table I, the single additional doses which require to be given after different numbers of fractions and gaps to achieve a particular end-point are set out for the groups of regimes (1) (ii), (b) (ii) and (c) (ii). It was shown earlier that that same end-point described by a CRE of 2160 can also be achieved by the simple regular schedule:

$$[6600\ (22/30)] = [(300)\ 22/30],$$

which follows the pattern of the above regimes before the gaps. If $N_1$ fractions, as recorded in Table II, are given before the gap which could be considered as being introduced into the above simple schedule, and if the total number of fractions, 22, remains unchanged, the number of fractions, $N_2$, to be given after the gap is:

$$N_2 = 22 - N_1$$

Therefore, for the same interfraction spacing of daily fractionation, the effective second schedules following the gap for Regimes (a) (ii), (b) (ii) and (c) (ii) must take the respective forms:

(a) (ii) $[D_2\ (18/26)]$
(b) (ii) $[D_2\ (13/19)]$
(c) (ii) $[D_2\ (8/12)]$

where $D_2$ is the total dose given in these schedules. In the terminology of Equation (3), each of these schedules achieves the CRE, $R_2$, which is numerically equivalent to the single additional doses, *d*, listed in Table I. Using Equation (1), the dose, $D_2$, to be given on each of the regimes (a) (ii), (b) (ii) and (c) (ii) for non-zero gaps can be simply calculated and are recorded in Table III. The effective additional

TABLE III.—*Total doses* ($D_2$) *required in 300rad/fraction schedules after gaps to compensate for decay of CRE*

| G | Regime (a)(ii) | (b)(ii) | (c)(ii) |
|---|---|---|---|
| 3 | — | 4480 | — |
| 7 | 5655 | 5115 | 4385 |
| 14 | 5955 | 5525 | 4655 |

doses, A, required to compensate for gaps after the nominal schedules; (a) 4 × 300 rad, (b) 9 × 300 rad and (c) 14 × 300 rad; can be found respectively from:

(a) $A = 1200 + D_2 - 6600$
(b) $A = 2700 + D_2 - 6600$
(c) $A = 4200 + D_2 - 6600$, and are summarized in Table IV. These results can be more clearly visualized from Fig. 5 in

TABLE IV.—*Effective additional doses* (*A*) *required to compensate for decay of CRE. (See text)*

| G | Regime (a)(ii) | (b)(ii) | (c)(ii) |
|---|---|---|---|
| 3 | — | 580 | — |
| 7 | 255 | 1215 | 1985 |
| 14 | 555 | 1625 | 2255 |

the main text of this paper, where their implications are compared with other relevant data and where the relevance of these studies on the effect of intervals in treatment to clinical practice and future efforts is fully explored and reviewed.